# Supplementary material for: MetaQTL: a package of new computational methods for the meta-analysis of QTL mapping experiments
Source: BMC Bioinformatics. 2007 Feb 8;8:49. doi: 10.1186/1471-2105-8-49 (PMC1808479; doi:10.1186/1471-2105-8-49)
Supplement: Additional File 7 — MetaQTL Package : jar file and tutorial. This Zip archive contains both the MetaQTL JAR file and the files of the tutorial. [file 1471-2105-8-49-S7.zip › org.inra.metaqtl/doc/org/metaqtl/algo/MetaMapAlgorithm.html]

MetaMapAlgorithm


|  |  |  |
| --- | --- | --- |
| **Overview** \| **Package** \| **Class** \| **Use** \| **Tree** \| **Deprecated** \| **Index** \| **Help** | |  |
| **PREV CLASS**   **NEXT CLASS** | **FRAMES**    **NO FRAMES**     **All Classes** |  |
| SUMMARY: NESTED \| FIELD \| CONSTR \| METHOD | DETAIL: FIELD \| CONSTR \| METHOD |  |


---


## org.metaqtl.algo Class MetaMapAlgorithm

```
java.lang.Object
  org.metaqtl.algo.MetaAlgorithm
      org.metaqtl.algo.MetaMapAlgorithm
```

**All Implemented Interfaces:**: IMetaAlgorithm

---

``` public class MetaMapAlgorithm extends MetaAlgorithm ```

This class is dedicated to the construction of a consensus
genetic map from a set of genetic maps based on a Weigthed
Least Square approach.

---

**Field Summary**

**Fields inherited from class org.metaqtl.algo.MetaAlgorithm**

`logger, loggerUp, workAmount, workProgress`


**Constructor Summary**

`MetaMapAlgorithm(IBioGenome[] maps,
IBioGenome skeleton,
IBioLocus[] dubious,
MetaDico mrkDico)`


| **Method Summary** | |
| --- | --- |
| `protected  MetaChrom` | `buildMetaChrom(ChromCluster cluster)`             For a cluster of chromosomes `cluster` this method builds the consensus chromosome using a Weighted Least Square strategy and returns the result as a `MetaChrom` object. |
| `MetaMap` | `getResult()` |
| `void` | `run()`             This method builds a consensus genetic map from the given array of `maps`. |
| `void` | `setMarkerDico(MetaDico mrkDico)` |
| `void` | `setMrkThreshold(int mrkThresh)` |

**Methods inherited from class org.metaqtl.algo.MetaAlgorithm**

`disableLogger, getLogger, getWorkAmount, getWorkProgress, isLoggerEnable, setLogger, setLogger`

**Methods inherited from class java.lang.Object**

`clone, equals, finalize, getClass, hashCode, notify, notifyAll, toString, wait, wait, wait`

**Constructor Detail**

### MetaMapAlgorithm

```
public MetaMapAlgorithm(IBioGenome[] maps,
                        IBioGenome skeleton,
                        IBioLocus[] dubious,
                        MetaDico mrkDico)
```

**Parameters:**: `maps` -: `skeleton` -: `dubious` -: `mrkDico` -


**Method Detail**

### getResult

```
public MetaMap getResult()
```

:   **Returns:**

---


### run

```
public void run()
```

:   This method builds a consensus genetic map from the given
    array of `maps`. The consensus map is returned
    as a `MetaMap`. If `skeleton` is not null
    then the positions of the markers on this map are assumed to
    be known and are used as anchor points in the construction
    of the consensus map. If `dubious is no null or not
    empty then the loci of this array won't be included in the consensus
    map.

    :   Parameters:: maps - the array of genetic maps.: skeleton - a genetic map used as skeleton.: dubious - an array of dubious loci to not include in the construction.`

---


### buildMetaChrom

```
protected MetaChrom buildMetaChrom(ChromCluster cluster)
```

:   For a cluster of chromosomes `cluster` this method
    builds the consensus chromosome using a Weighted Least Square
    strategy and returns the result as a `MetaChrom` object.

    :   **Parameters:**: `cluster` - the cluster of chromosomes. **Returns:**: the consensus chromosome.

---


### setMarkerDico

```
public void setMarkerDico(MetaDico mrkDico)
```

:   **Parameters:**: `mrkDico` -

---


### setMrkThreshold

```
public void setMrkThreshold(int mrkThresh)
```

:   **Parameters:**: `mrkThresh` -


---


|  |  |  |
| --- | --- | --- |
| **Overview** \| **Package** \| **Class** \| **Use** \| **Tree** \| **Deprecated** \| **Index** \| **Help** | |  |
| **PREV CLASS**   **NEXT CLASS** | **FRAMES**    **NO FRAMES**     **All Classes** |  |
| SUMMARY: NESTED \| FIELD \| CONSTR \| METHOD | DETAIL: FIELD \| CONSTR \| METHOD |  |


---
